# Impact of Neoadjuvant Immunochemotherapy on Surgical Outcomes in Management of Early Triple‐Negative Breast Cancer

**DOI:** 10.1155/tbj/5534220

**Published:** 2026-07-17

**Authors:** Tamasine Khadrouche, Mathilde Morisseau, Gabrielle Selmes, Charlotte Vaysse, Camille Franchet, Carole Massabeau, Mony Ung, Eleonora De Maio, Jean-Louis Lacaze, Eva Jouve

**Affiliations:** ^1^ Department of Surgical Oncology, Claudius Regaud Institute–IUCT Oncopole, Toulouse, France; ^2^ Biostatistics & Health Data Science Unit, Claudius Regaud Institute–IUCT Oncopole, Toulouse, France; ^3^ Department of Surgical Oncology, University Hospital Center Toulouse-IUCT Oncopole, Toulouse, France; ^4^ Department of Pathology, Claudius Regaud Institute–IUCT Oncopole, Toulouse, France; ^5^ Department of Radiotherapy, Claudius Regaud Institute–IUCT Oncopole, Toulouse, France; ^6^ Department of Medical Oncology, Claudius Regaud Institute – IUCT Oncopole, Toulouse, France

**Keywords:** immune-related adverse events, neoadjuvant chemotherapy, neoadjuvant immunochemotherapy, surgical outcomes, triple-negative breast cancer

## Abstract

**Background:**

Triple‐negative breast cancer (TNBC) is an aggressive subtype with poor prognosis. Neoadjuvant immunochemotherapy (NAIC) combining chemotherapy and pembrolizumab is now standard for Stage II‐III TNBC. This study evaluates the impact of NAIC on surgical outcomes versus neoadjuvant chemotherapy (NAC).

**Methods:**

A retrospective cohort study included 117 Stage II‐III TNBC patients treated with NAIC or NAC (October 2019–May 2023). Surgical complications, time to surgery, time to radiotherapy, and pathologic response were assessed. Complications were classified using the Clavien–Dindo scale; immune‐related adverse events (irAEs) followed the 2017 CTCAE criteria.

**Results:**

Among 117 patients, 59 received NAIC and 58 NAC. Chemotherapy‐related adverse events were similar (NAIC: 78.0%, NAC: 75.9%). irAEs occurred in 50.8% of NAIC patients, with 8.5% experiencing severe irAEs. Surgical complications were more frequent in NAIC (27.1%) than NAC (17.2%), though not statistically significant; seroma was the most common. Delays > 12 weeks in initiating radiotherapy were more frequent in NAIC (10.7%) than NAC (0%).

**Conclusion:**

NAIC did not significantly increase postoperative complications compared to NAC. However, irAEs and potential treatment delays warrant careful management. Despite these risks, NAIC remains a viable option for early TNBC with manageable surgical outcomes.

## 1. Introduction

Triple‐negative breast cancer (TNBC) is the least frequent subtype of early breast cancer, accounting for 10%–15% of cases. TNBC is characterized by the absence of estrogen and progesterone receptors and the absence of HER2 overexpression and/or gene amplification [1]. TNBC is generally more aggressive, with earlier onset and higher rates of distant recurrence and mortality within the first 5 years compared to other breast cancer subtypes. Even after adjusting for age, stage, grade, and adjuvant chemotherapy, early TNBC has worse overall survival compared to hormone receptor–positive/HER2‐ breast cancers [2, 3].

The KEYNOTE‐522 trial demonstrated that the addition of pembrolizumab to neoadjuvant chemotherapy (NAC) significantly improved pathologic complete response (pCR) rates, event‐free survival, and overall survival at 60 months compared to NAC alone [4]. Consequently, neoadjuvant immunochemotherapy (NAIC) has become the standard treatment for Stage II‐III TNBC [5, 6]. However, this regimen, which includes immunotherapy and carboplatin, often results in frequent adverse events [7]. Patients treated in the KEYNOTE trials and other neoadjuvant immunotherapy studies experienced a wide range of immune‐related adverse events (irAEs) [8–11]. Despite the well‐established surgical risk factors such as obesity, smoking history, and diabetes [12–14], the impact of irAEs on surgical outcomes has received limited attention.

This retrospective single‐center study aimed to investigate surgical outcomes, including surgical complications, time to surgery, and time to radiotherapy, in patients undergoing NAIC compared to those receiving NAC for Stage II‐III TNBC.

## 2. Materials and Methods

A retrospective query was made on the electronic medical record of our institution for all consecutive patients undergoing neoadjuvant treatment between October 2019 and May 2023: initially with chemotherapy alone, then combined with pembrolizumab starting in April 2022 (the date of the favorable reimbursement decision in France for this indication). Patients enrolled in clinical trials or undergoing surgery at other institutions were excluded. The study was approved by the National and Institutional Review Boards (no. F20240119104231).

Neoadjuvant treatment in the NAIC group followed the KEYNOTE‐522 regimen, consisting of four cycles of pembrolizumab associated with paclitaxel and carboplatin followed by four cycles of pembrolizumab plus anthracycline (epirubicin) and cyclophosphamide (EC). Neoadjuvant treatment in the NAC group consisted of EC followed by paclitaxel. Surgery was scheduled 4–6 weeks after EC–pembrolizumab in NAIC protocol and 2–4 weeks after paclitaxel in NAC protocol, according to our institutional recommendations. Surgical management of the breast was decided with clinical and iconographic evaluation before and after neoadjuvant treatment. According to our institutional recommendations, surgical axillary management was defined by the initial nodal status: sentinel lymph node dissection (SLND) for cN0 initial status and axillary dissection (AD) for cN1 initial status. Radiotherapy was scheduled as early as possible after surgery.

Data regarding relevant medical and life history were recorded. Neoadjuvant treatment complications were classified according to the Common Terminology Criteria for Adverse Events (CTCAE v5.0) Version 5. Severe irAEs included all irAEs of ≥ Grade 3.

Any surgical complication occurring before initiation of radiotherapy was rated with the Clavien–Dindo Classification [15]. Only Grade II–V complications were analyzed. Complications requiring surgical, endoscopic, or radiological intervention were classified as Grade III (IIIa not under general anesthesia, IIIb under general anesthesia). Severe complications included all surgical complications ≥ Grade III. Time to surgery, defined as time from the last preoperative chemotherapy to surgery, and time to radiotherapy, defined as time from surgery to the first fraction of radiotherapy, were reviewed. A more than 42‐day (6‐week) time between the end of chemotherapy and surgery was considered as excessive. A more than 84‐day (12‐week) time between surgery and radiotherapy was considered as excessive. pCR was defined as no residual invasive cancer in the breast or the axillary nodes (ypT0 ypN0 or ypTis ypN0). The rate of cN1 initial status becoming yN0 was described.

### 2.1. Statistical Analysis

Patient information was extracted from the IUCT‐O electronic medical records. Population characteristics were described by median, minimum, and maximum for quantitative variables and by frequencies and percentages of each modality for qualitative variables. Comparisons between groups were assessed using the chi‐square test or Fisher’s exact test for qualitative variables and the Kruskal–Wallis test for quantitative variables. Multivariate analyses were performed using a logistic regression model, and odds ratios (ORs) were estimated with their 95% confidence intervals. All statistical tests were two‐sided, and a *p* value < 0.05 was considered as statistically significant. All statistical analyses were conducted using Stata Version 18 software (StataCorp LLC, College Station, TX).

## 3. Results

### 3.1. Patient and Tumor Characteristics

Out of 117 patients meeting the inclusion criteria, 59 were treated with NAIC and 58 with NAC. Patient and tumor characteristics were similar between groups, including rates of obesity (BMI ≥ 30), smoking history, and relevant medical histories (Table 1). Invasive ductal carcinoma was the predominant histological subtype (94.9%), with T2 tumors accounting for the majority (NAIC: 66.1%, NAC: 65.5%). Half of the patients presented with axillary involvement at diagnosis (NAIC: 50.8%, NAC: 50.0%).

**TABLE 1:** Patient and tumor characteristics.

	Total (*n* = 117)	NAIC (*n* = 59)	NAC (*n* = 58)	*p* value
Median age at diagnosis (range) (*n* = 117)	50 (26–79)	48 (30–74)	50.5 (26–79)	0.520
BMI (*n* = 117)				
< 30	96 (82.1%)	47 (79.7%)	49 (84.5%)	0.497
≥ 30	21 (17.9%)	12 (20.3%)	9 (15.5%)	
Bra size (n = 79)				0.464
A–C	53 (67.1%)	25 (71.4%)	28 (63.6%)	
D–F	26 (32.9%)	10 (28.6%)	16 (36.4%)	
Smoking status (*n* = 108)				0.434
No	80 (74.1%)	36 (70.6%)	44 (77.2%)	
Yes	28 (25.9%)	15 (29.4%)	13 (22.8%)	
Relevant medical history (*n* = 117)				
High blood pressure	13 (11.1%)	7 (11.9%)	6 (10.3%)	0.794
Diabetes	8 (6.8%)	7 (11.9%)	1 (1.7%)	0.061
Cardiovascular history	4 (3.4%)	3 (5.1%)	1 (1.8%)	0.619
Breast cancer history	6 (5.1%)	1 (1.7%)	5 (8.6%)	0.114
Radiotherapy history	6 (5.1%)	1 (1.7%)	5 (8.6%)	0.114
Status T (*n* = 117)				
T1	5 (4.3%)	1 (1.7%)	4 (6.9%)	
T2	77 (65.8%)	39 (66.1%)	38 (65.5%)	
T3	23 (19.7%)	13 (22.0%)	10 (17.2%)	
T4	12 (10.2%)	6 (10.2%)	6 (10.3%)	
Status N (*n* = 117)				0.927
N0	58 (49.6%)	29 (49.2%)	29 (50.0%)	
*N*+	59 (50.4%)	30 (50.8%)	29 (50.0%)	
Histology (*n* = 117)				
Ductal	111 (94.9%)	55 (93.2%)	56 (96.6%)	
Lobular	1 (0.9%)	1 (1.7%)	0 (0.0%)	
Others	5 (4.3%)	3 (5.1%)	2 (3.4%)	

### 3.2. Medical and Surgical Treatment

A total of 90 patients (76.9%) experienced chemotherapy‐related adverse events (crAEs), with comparable rates between NAIC (78.0%) and NAC (75.9%) groups. Severe crAEs (≥ Grade 3) were more frequent in the NAIC group (16.9% vs. 6.9%). No significant difference was observed in terms of chemotherapy discontinuation (NAIC *n* = 9, 15.5%; NAC *n* = 12, 21.1%, *p* = 0.442).

irAEs were reported in 50.8% of patients in the NAIC group, with 8.5% (*n* = 5) suffering severe irAEs (≥ Grade 3). Several patients experienced more than one irAE. The most common irAEs were endocrine disorders such as thyroiditis (*n* = 13), adrenal insufficiency (*n* = 7), and diabetes (*n* = 1). Three episodes of pneumonitis, 3 episodes of dermatitis, 2 episodes of anaphylaxis, 2 episodes of hepatitis, 2 episodes of arthritis, 2 episodes of colitis, one myocarditis, and one hypophysitis were also observed. Severe irAEs included Grade 3 adrenal insufficiency (*n* = 2), Grade 3 colitis (*n* = 2), and Grade 4 diabetes (*n* = 1). Pembrolizumab was discontinued in 40.7% of NAIC patients, mainly due to toxicity.

Breast‐conserving surgery was the most commonly performed surgery (63.2%), with comparable frequencies observed between the NAIC and NAC groups. AD was carried out in 59.5% of patients, with similar rates between the NAIC and NAC groups (Table 2).

**TABLE 2:** Treatment characteristics.

	Total (*n* = 117)	NAIC (*n* = 59)	NAC (*n* = 58)	*p* value
Chemotherapy adverse events (*n* = 117)	90 (76.9%)	46 (78.0%)	44 (75.9%)	0.787
Severe chemotherapy AE (≥ 3)	14 (12.0%)	10 (16.9%)	4 (6.9%)	
Reasons for premature chemotherapy discontinuation (*n* = 115)				
Number of discontinuations	21 (18.3%)	9 (15.5%)	12 (21.1%)	0.442
Toxicity	14 (70.0%)	6 (75.0%)	8 (66.7%)	
Progression	2 (10.0%)	0 (0.0%)	2 (16.7%)	
Other	4 (20.0%)	2 (25.0%)	2 (16.7%)	
Immunotherapy adverse events (*n* = 59)		30 (50.8%)		
Severe immunotherapy AE (≥ 3)		5 (8.5%)		
Reasons for premature immunotherapy discontinuation (n = 59)				
Number of discontinuations		24 (40.7%)		
Toxicity		20 (83.3%)		
Progression		1 (4.2%)		
Other		3 (12.5%)		
Breast surgery (*n* = 117)				0.303
Breast conservation	74 (63.2%)	40 (67.8%)	34 (58.6%)	
Mastectomy	43 (36.8%)	19 (32.2%)	24 (41.4%)	
*IBR*	21 (48.8%)	9 (47.4%)	12 (50.0%)	
Implant	18 (85.7%)	8 (88.9%)	10 (83.3%)	
Flap	3 (14.3%)	1 (11.1%)	2 (16.7%)	
Axillary surgery (n = 116)				0.732
SLND	47 (40.5%)	23 (39.0%)	24 (42.1%)	
AD	69 (59.5%)	36 (61.0%)	33 (57.9%)	

Abbreviations: AD, axillary dissection; IBR, immediate breast reconstruction; SLND, sentinel lymph node dissection.

### 3.3. Surgical Outcomes

#### 3.3.1. Surgical Complications

**FIGURE 1:**
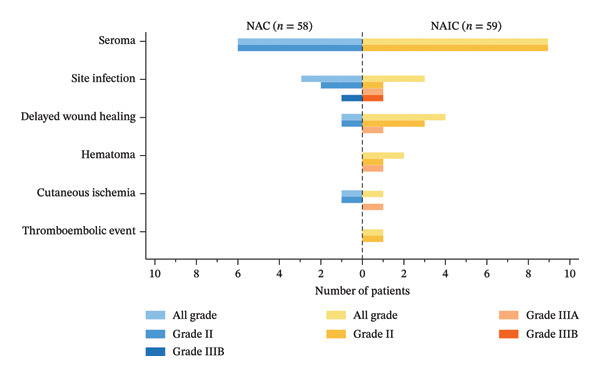
Grade II–V Clavien–Dindo surgical complications by type. Some patients experienced more than one complication.

Grade II–V Clavien–Dindo surgical complications occurred in 22.2% of patients, with a higher but nonsignificant rate in the NAIC group (27.1% vs. 17.2%, *p* = 0.199) (Table 3). Thirty‐one events were diagnosed, as some patients experienced more than one complication (Figure 1). The most frequent complication was seroma (48.4%), followed by postoperative infections (19.4%) and delayed wound healing (16.1%). Severe complications (≥ Grade III) were rare: 4 patients (6.8%) in the NAIC group vs. 1 patient (1.7%) in NAC group. No Grade IV complications were observed. Excluding seroma, 8 patients (13.6%) in the NAIC group and 4 patients (6.9%) in the NAC group suffered from at least one Grade II–V Clavien–Dindo early postoperative complication (*p* = 0.235). Regarding reconstructions, implant removal was required in 2 of the 8 patients in the NAIC group and in 1 of the 10 patients in the NAC group. The outcomes of the three flap‐based reconstructions were satisfactory.

**TABLE 3:** Clavien–Dindo surgical complications by patients.

	Total (*n* = 117)	NAIC (*n* = 59)	NAC (*n* = 58)	*p* value
Surgical complications ≥ Grade II	26 (22.2%)	16 (27.1%)	10 (17.2%)	0.199
• Seroma	15 (12.8%)	9 (15.3%)	6 (10.3%)	
• Site infection	6 (5.1%)	3 (5.1%)	3 (5.2%)	
• Delayed wound healing	5 (4.3%)	4 (6.8%)	1 (1.7%)	
• Hematoma	2 (1.7%)	2 (3.4%)	0 (0.0%)	
• Cutaneous ischemia	2 (1.7%)	1 (1.7%)	1 (1.7%)	
• Thromboembolic events	1 (0.9%)	1 (1.7%)	0 (0.0%)	

Surgical complications ≥ Grade II excluding seroma	12 (13.6%)	8 (13.6%)	4 (6.9%)	0.235

Severe surgical complications (≥ Grade III)	5 (4.3%)	4 (6.8%)	1 (1.7%)	

#### 3.3.2. Univariate and Multivariate Analysis

AD was associated with increased surgical complications in both univariate and multivariate analyses (OR = 4.26 [1.29; 14.02]; *p* = 0.017). Neither type of neoadjuvant treatment nor type of breast surgery was associated with surgical complications in univariate or multivariate analysis. None of the analyzed preexisting factors were associated with surgical complications, even though BMI over 30 had a tendency toward significance (Table 4). We aimed to assess whether severe irAEs led to more surgical complications, but the low number of events did not allow us to draw any conclusions. Six patients out of the seven diagnosed with immune‐related adrenal insufficiency supplemented by corticotherapy suffered from surgical complications; however, no patient had an over 6‐week time to surgery.

**TABLE 4:** Univariate and multivariate analysis of Grade II–V surgical complications.

	Univariate analysis			Multivariate logistic regression^∗^	
	No	Yes	*p* value	OR [95% CI]	*p* value
	(*n* = 91)	(*n* = 26)		(*n* = 116)	
Neoadjuvant treatment (n = 117)			0.199		
NAC	48 (52.7%)	10 (38.5%)		1.00	
NAIC	43 (47.3%)	16 (61.5%)		1.76 [0.69; 4.52]	0.239
BMI (n = 117)			*0.079*		
< 30	78 (85.7%)	18 (69.2%)		1.00	
≥ 30	13 (14.3%)	8 (30.8%)		2.24 [0.76; 6.58]	0.141
Smoking history (n = 108)			0.606		
No	62 (72.9%)	18 (78.3%)			
Yes	23 (27.1%)	5 (21.7%)			
Breast surgery (n = 117)			0.112		
Mastectomy	30 (33.0%)	13 (50.0%)		1.00	
BCS	61 (67.0%)	13 (50.0%)		0.67 [0.26; 1.75]	0.414
Axillary surgery (n = 116)			**0.003**		**0.017**
SLND	43 (47.8%)	4 (15.4%)		1.00	
AD	47 (52.2%)	22 (84.6%)		4.26 [1.29; 14.02]	
IBR (n = 112)			0.243		
No	73 (83.9%)	18 (72.0%)			
Yes	14 (16.1%)	7 (28.0%)			
High BP (n = 117)			0.730		
No	80 (87.9%)	24 (92.3%)			
Yes	11 (12.1%)	2 (7.7%)			

*Note:* Significant values are given in bold (< 0.05).

Abbreviations: AD, axillary dissection; BCS, breast conservation surgery; BP, blood pressure; SLND, single lymph node dissection.

^∗^Variables with *p* value < 0.020 in univariate analysis.

#### 3.3.3. Time to Locoregional Treatment

Median time to surgery was significantly longer in the NAIC group (25.0 days vs. 18.0 days, *p* < 0.001), with 5.1% of NAIC patients exceeding 42 days compared to 3.4% in the NAC group. Median time to radiotherapy was also slightly longer in the NAIC group (56 days vs. 53.5 days, *p* = 0.044), with 10.7% of NAIC patients experiencing delays beyond 84 days compared to none in the NAC group (*p* = 0.027) (Table 5). Among the six patients with a delay of more than 84 days, the circumstances and causes attributable to the delay varied: 2 surgical, 1 medical, and 1 organizational constraints and 2 mixed (Table 6). There was no significant correlation between radiotherapy delays and the occurrence of crAE (56.0 days with crAE, 55.0 days without crAE, *p* = 0.819) or surgical complications (55.5 days in both groups).

**TABLE 5:** Time to locoregional treatment.

	Total (*n* = 117)	NAIC (*n* = 59)	NAC (*n* = 58)	*p* value
Time to surgery (days)				
• Median (range)	22.0 (9.0; 79.0)	25.0 (19.0; 54.0)	18.0 (9.0; 79.0)	< 0.001
• ≥ 42 days *n* (%)	5 (4.3%)	3 (5.1%)	2 (3.4%)	
Time to radiotherapy (days)				
• Median (range)	55.5 (25.0; 161.0)	56.0 (39.0; 161.0)	53.5 (25.0; 83.0)	0.044
• ≥ 84 days *n* (%)	6 (5.5%)	6 (10.7%)	0 (0.0%)	0.027

**TABLE 6:** Details of patients with delay to radiotherapy ≥ 84 days.

	Radiation delay (days)	Adjuvant immunotherapy	Chemotherapy AE^∗^ type and grade	irAE type and grade	Surgical complication^∗∗^ type and grade	Attributable cause of the delay
Patient 1	90	Yes	Hematotoxicity gr II	No	No	Organizational constraints
Patient 17	92	No	Hematotoxicity gr I Febrile neutropenia gr III	Thyroiditis gr II Adrenal insufficiency gr III Colitis gr III	Site infection gr IIIb	Surgical complication + irAE
Patient 19	104	No	Anaphylaxis gr II Hematotoxicity	Anaphylaxis gr II	Delayed wound healing gr II	Surgical complication
Patient 24	161	No	Nausea gr II Hematotoxicity gr III Infection gr III	Thyroiditis gr II Adrenal insufficiency gr II	Delayed wound healing gr IIIa Site infection gr II Cutaneous ischemia gr IIIa	Surgical complication
Patient 25	102	No	Dermatotoxicity gr I	Hepatitis gr II Thyroiditis gr II	No	Organizational constraints + irAE
Patient 53	86	Yes	Anaphylaxis gr I	No	Seroma gr I	Organizational constraints

*Note:* Neoadjuvant treatment complications classified according to CTCAE v5.0.

^∗^All toxic effects of chemotherapy occurred and ended before surgery.

^∗∗^Clavien–Dindo Grade III complications required surgical interventions, performed either without general anesthesia (IIIa) or under general anesthesia (IIIb).

Delays to radiotherapy were not statistically different between patients with or without irAEs (56.0 days vs. 58.0 days, *p* = 0.67). Among the five patients with severe irAEs, one experienced a time to surgery longer than 42 days, and one had a time to radiotherapy longer than 84 days, compared to two and five patients, respectively, among the 54 patients without severe irAEs.

#### 3.3.4. Pathologic Response

There was a nonsignificant increase in pCR (breast and axilla) in the NAIC group (NAIC 64.4%; NAC 56.1%, *p* = 0.363). Among the 59 patients with initial cN + status, conversion to ypN0 occurred in 78.0% of them, 73.3% of the NAIC group and 82.8% of the NAC group.

## 4. Discussion

This retrospective single‐center study found a higher rate of postoperative Grade II–V Clavien–Dindo complications after NAIC than after NAC, but without statistical significance (NAIC: 27.1%; NAC: 17.2%; *p* = 0.199), even after adjusting for BMI, type of breast surgery, and type of axillary surgery. In both groups, the most common surgical complication was Grade II seroma. Severe breast surgery complications requiring return to theater, i.e., Grade III, were infrequent. They were more numerous with NAIC than with NAC (*NAIC n = 4; NAC n = 1*), but the small number of complications does not allow any conclusions to be drawn.

As expected, AD increased the risk of postoperative Grade II–V complications in this study, both in univariate analysis and after adjustment for neoadjuvant treatment, BMI, and type of breast surgery. Indeed, AD is known to result in more seromas, axillary infections, lymphedema, and paresthesia than SLND, independently of neoadjuvant treatment [16, 17]. Although obesity and smoking history are well‐known risk factors for infectious and wound‐healing complications in breast surgery (12–14), smoking history (active or weaned) was not significantly associated with surgical complications in our study, and we only observed a tendency for obesity.

A recent American retrospective single‐arm study published by Woodfin et al. on perioperative outcomes in 87 patients treated by NAIC for early TNBC reported a similar postoperative complication rate of 24.1% [18]. In another study published by Myers et al., surgical outcomes were compared in a cohort of 143 patients undergoing NAIC with the KEYNOTE‐522 regimen and 287 patients receiving NAC. Postoperative complications occurred in 7.9% patients undergoing NAIC and 9.1% patients undergoing NAC [19]. These different results might be explained by the different definitions used to describe surgical complications as well as by the different data collection periods. Seroma accounted for half of the surgical complications in our study, whereas they were not included in Myers et al.’s study. Patient and neoplastic characteristics also differed between the cohorts with a higher rate of SLND in the review by Myers et al. Indeed, the 59.5% AD rate in our study is similar to that of Woodfin et al. but more than twice the rate of Myers et al., i.e., 33% and 21% in the NAIC and NAC groups, respectively. Apart from seroma, Grade II–V surgical complications, mainly delayed wound healing and site infection, were more frequent in the NAIC group (*n* = 8, 13.6%) than in the NAC group (*n* = 4, 6.9%) but without statistical significance (*p* = 0.235) in our study. This low rate of complications, excluding seroma, is reassuring with regard to the care pathway for patients, particularly for reconstructed patients (36.8% of mastectomy patients in our study) and in comparison with major studies on the subject [20, 21]. Larger sample sizes would, of course, be required to validate these findings, especially in relation to the different reconstruction modalities (flap or implant‐based, prepectoral or subpectoral, with or without a matrix, and with or without nipple‐areola complex preservation). Breast surgery complications after NAC have been reported with results close to our 17.2% complication rate [22, 23].

In our study, 50.8% of patients under NAIC experienced irAEs, with 8.5% suffering from severe irAEs. Adrenal insufficiency was diagnosed in 12.5% of patients, all treated with corticosteroids during surgery, and six patients had surgical complications. In the KEYNOTE‐522 trial, 33.4% of patients had irAEs, 12.9% of which were Grade ≥ 3, and 2.6% had adrenal insufficiency. As immunotherapy combined with chemotherapy becomes standard care, underdiagnosed irAEs can have significant perioperative consequences. The Society for Immunotherapy of Cancer recommends monitoring for diabetes, adrenal insufficiency, and thyroid dysfunction in patients treated with pembrolizumab [24]. Our institution applies specific anesthetic vigilance, including detailed biological tests and attention to autoimmune symptoms, emphasizing the importance of initial patient evaluation and ongoing clinical vigilance throughout oncologic treatment.

After NAC, surgery is typically scheduled to avoid the period of risk for aplasia and postoperative infection [23, 25]. Because cell count recovery is faster after paclitaxel than anthracycline‐based regimens, surgery can be offered earlier in the NAC group than in the NAIC group, depending on the type of chemotherapy protocol. Therefore, median time to surgery was significantly one week longer with NAIC than with NAC (25 days vs. 18 days, respectively). Rached et al. reported a similar delay of 22 days in their 100‐patient NAIC study, which is shorter than the 32‐day delay observed by Myers et al. [26, 27]. Moreover, despite the addition of irAEs and the seemingly more important Grade III surgical complications in the NAIC group, the proportion of patients with over a 42‐day time to surgery was comparable in both groups.

Time to radiotherapy was similar in both groups (*NAIC: 56 days; NAC: 53.5 days,* *p* = 0.044). With a 2.5‐day increased median time to radiotherapy in the NAIC group, this difference does not seem clinically pertinent. However, a significant over 84‐day time to radiotherapy was observed in 6 patients (10.7%) in the NAIC group versus none in the NAC group, but no correlation with occurrences of crAEs or surgical complications was found to explain the observed difference, perhaps due to a lack of statistical power. Contrary to the study by Myers et al. [27], experiencing an irAE was not associated with time to radiotherapy. Hershman et al. found a decline in overall survival when radiotherapy was administered after 12 weeks [28], but our follow‐up is too short and the cohort is too small to observe such a carcinologic impact.

On the final pathological report, there was a higher though nonsignificant pCR rate of 64.4% in the NAIC group compared to 56.1% in the NAC group. The Phase III KEYNOTE‐522 trial showed a 64.8% pCR rate in the pembrolizumab plus chemotherapy group compared to 51.2% in the control group at final analysis [8]. The higher pCR rate in our control group compared to the control group in the KEYNOTE‐522 study may be explained by the use of a dose‐dense regimen, which is more effective than the KEYNOTE‐522 control arm [29]. With a large rate of conversion from cN + to ypN0 (78%), axillary downscaling with targeted axillary dissection (TAD) is now accepted in our institution on the basis of international recommendations [5, 30, 31].

### 4.1. Strengths

This real‐life study is the first French consecutive and retrospective study on surgical outcomes of NAIC. Findings are in line with the literature. The perioperative complication rate and time to surgery and radiotherapy were similar to those of recent real‐life studies on NAIC. The study groups were statistically comparable, allowing for a robust and accurate analysis throughout the study.

### 4.2. Limitations

Our study has several limitations inherent to its retrospective, single‐center design, including an increased risk of selection and information biases. The chemotherapy regimens also varied between the treatment arms, which affected the time from chemotherapy to surgery. Furthermore, the small sample size (*n* = 117) limits its statistical power, potentially hindering the detection of clinically significant differences, especially regarding rare events such as severe surgical complications or high‐grade irAEs. To partially address these limitations, we performed multivariate analyses accounting for major confounding factors identified in the literature (BMI, diabetes, smoking history, and type of surgical procedure). Despite these adjustments, our findings should be considered exploratory. Further prospective multicenter studies with larger patient cohorts are required to validate our conclusions.

## 5. Conclusion

In this retrospective study, no statistically significant difference was found in the rate of postoperative complications after NAIC or NAC. Despite a high incidence of irAEs in the NAIC group, the surgical management of patients treated with immunotherapy appears feasible, with no major safety concerns observed.

## Funding

No funding was received for this manuscript.

## Conflicts of Interest

The authors declare no conflicts of interest.

## Data Availability

Data are available on request due to privacy/ethical restrictions.
